# The Type Writer

**Published:** 1874-11

**Authors:** 


### THE TYPE WRITER.

ONE of the publishers of HALL'S JOURNAL OF HEALTH, in passing down Broadway the other day, joined a crowd of persons who were looking in at a window. Inside were a number of individuals of both sexes surrounding what appeared to be a new kind of sewing machine. He stepped in and observed. Seated at the machine was a young man who was playing upon a series of keys, very much as a piano is played upon. The keys were merely little round glass knobs, and inside of each knob, and protected by the glass, was a letter of the alphabet, or a numeral or a punctuation mark. There were unoccupied machines in the room, and the inquirer left the crowd around the operator and approached one of these. It is a graceful little lacquered metallic box when closed, which, when opened, reveals four banks of these little lettered key knobs. The box is on a table which is not unlike a sewing machine table. There is a treadle, but it is used only to throw back, at the end of each line, a roller having the paper on it. An attendant approached and asked if we would like to see the machine work. We said we should, and then proceeded to say that we would like to ask how long it would take a person to learn to operate it rapidly; how it compared, for rapidity of execution, with the pen; whether purchasers generally found it advantageous to keep it in practical use, or looked upon it chiefly as a curiosity; and whether it seemed useful enough to commend it to general favor.

The attendant gave no verbal answer to this string of questions, but merely asked the name of the person whom he was addressing. We took out a copy of the JOURNAL OF HEALTH and pointed to the firm-name of the publishers. Meanwhile he had seated himself in front of the instrument, and in a moment he commenced running his fingers rapidly over the keys. In a half a minute or so he handed us a slip of paper, with the following neatly printed upon it:

"New York, Oct. 20, 1874.

"Messrs. E. H. GIBBS & Co., Publishers *Hall's Journal of Health*, No. 84 Broadway, New York.

"Gentlemen,—The price of this machine is $125. It requires but a few hours to learn to operate it rapidly, and those who once employ it in correspondence or for copying are unwilling to go without it. We believe it is destined to be almost universally employed."

It was clear to our mind that the operator could not have written these lines with pen or pencil in the few seconds which he occupied in recording them with the types of this machine. He then asked us if we would like to operate the machine. We expressed doubts of our ability, but were induced to make the attempt. Of course we could not write rapidly, as we had to look up the letters on the keys; but we were able in a brief space to produce the following :

"WE ENTIRELY AGREE WITH YOU IN YOUR ESTIMATE OF THE VALUE OF YOUR WONDERFUL MACHINE.

"By its use legible writing becomes a cleanly and delightful amusement, rather than a laborious and unpleasant task. We shall gladly say as much as this to the readers of HALL'S JOURNAL OF HEALTH.—Yours truly,

"E. H. GIBBS & Co., Publishers."

The operation of the machine is very simple, and the act of writing with it is extremely fascinating. If you desire to write manifold copies, you can do so by merely alternating the sheets of paper with carbon paper ; and the blow struck by the type on depressing the key gives twenty clear impressions. It is well calculated to supersede the use of the pen for nearly all purposes except book-keeping. The ink is supplied by a ribbon, and this renders inkstands unnecessary, and obviates ink stains upon the fingers and clothing and furniture and carpets. It works a decided revolution in correspondence, since it enables aged and infirm persons and children, and bad penmen, to write rapidly and legibly.

We were so well pleased with our first efforts in type-writing, that we asked permission to call again. This was cheerfully accorded us, and we propose to continue to practice on the machine until we can write at the rate of sixty words a minute—by no means a difficult feat, as we are assured. If any of our readers desire to learn more about this ingenious machine, they have only to communicate with the Publishers of this JOURNAL, by mail.

